# Human and great ape red blood cells differ in plasmalogen levels and composition

**DOI:** 10.1186/1476-511X-10-101

**Published:** 2011-06-17

**Authors:** Ann B Moser, Steven J Steinberg, Paul A Watkins, Hugo W Moser, Krishna Ramaswamy, Kimberly D Siegmund, D Rick Lee, John J Ely, Oliver A Ryder, Joseph G Hacia

**Affiliations:** 1 Hugo W. Moser Research Institute at Kennedy Krieger, and Department of Neurology, Johns Hopkins University School of Medicine, Baltimore, MD, 21205, USA; 2 Department of Biochemistry and Molecular Biology, Broad Center for Regenerative Medicine and Stem Cell Research, University of Southern California, Los Angeles, CA, 90089, USA; 3 Department of Preventive Medicine, University of Southern California, Los Angeles, CA, 90089, USA; 4 Washington National Primate Research Center, University of Washington, Seattle, WA, 98195, USA; 5 Alamogordo Primate Facility, New Mexico, NM 88330, USA; 6 Institute for Conservation and Research, Zoological Society of San Diego, Escondido, CA, 92027, USA

## Abstract

**Background:**

Plasmalogens are ether phospholipids required for normal mammalian developmental, physiological, and cognitive functions. They have been proposed to act as membrane antioxidants and reservoirs of polyunsaturated fatty acids as well as influence intracellular signaling and membrane dynamics. Plasmalogens are particularly enriched in cells and tissues of the human nervous, immune, and cardiovascular systems. Humans with severely reduced plasmalogen levels have reduced life spans, abnormal neurological development, skeletal dysplasia, impaired respiration, and cataracts. Plasmalogen deficiency is also found in the brain tissue of individuals with Alzheimer disease.

**Results:**

In a human and great ape cohort, we measured the red blood cell (RBC) levels of the most abundant types of plasmalogens. Total RBC plasmalogen levels were lower in humans than bonobos, chimpanzees, and gorillas, but higher than orangutans. There were especially pronounced cross-species differences in the levels of plasmalogens with a C16:0 moiety at the *sn*-1 position. Humans on Western or vegan diets had comparable total RBC plasmalogen levels, but the latter group showed moderately higher levels of plasmalogens with a C18:1 moiety at the *sn*-1 position. We did not find robust sex-specific differences in human or chimpanzee RBC plasmalogen levels or composition. Furthermore, human and great ape skin fibroblasts showed only modest differences in peroxisomal plasmalogen biosynthetic activity. Human and chimpanzee microarray data indicated that genes involved in plasmalogen biosynthesis show cross-species differential expression in multiple tissues.

**Conclusion:**

We propose that the observed differences in human and great ape RBC plasmalogens are primarily caused by their rates of biosynthesis and/or turnover. Gene expression data raise the possibility that other human and great ape cells and tissues differ in plasmalogen levels. Based on the phenotypes of humans and rodents with plasmalogen disorders, we propose that cross-species differences in tissue plasmalogen levels could influence organ functions and processes ranging from cognition to reproduction to aging.

## Background

**Figure 1:**
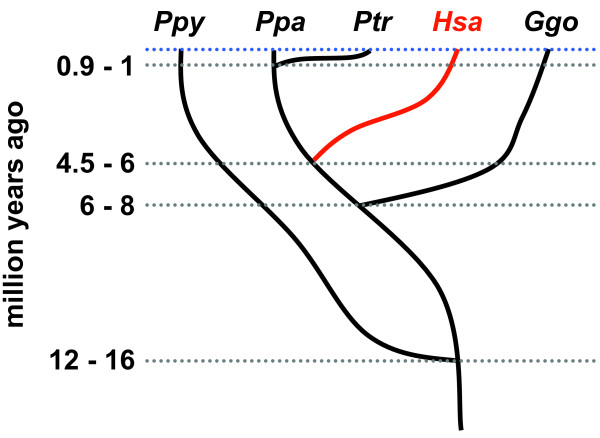
**Phylogenetic relationships of our cohort**. We provide a simplified overview of the phylogenies of the species examined in this study along with the inferred age of the last common ancestors at different branch points [8,9]. The present day is indicated by the light blue dotted line and the human lineage is highlighted in red. *Ppy *= *Pongo pygmaeus *(orangutan), *Ppa *= *Pan panisicus *(bonobo), *Ptr *= *Pan troglodytes*, *Hsa *= *Homo sapiens *(human), and *Ggo *= *Gorilla gorilla *(gorilla). Although we recognize the close relationships among all five species, we use the term 'great apes' in reference to chimpanzees, bonobos, gorillas, and orangutans.

Several decades after an early report that humans and Japanese macaques (*Macaca fuscata*) have different susceptibilities to atheromatosis [1], it was established that lipid metabolism and cardiovascular disease risks vary among human and nonhuman primates [2-5]. In agreement with phylogenetic relationships [6-9], human blood lipid profiles most closely resemble those of their closest living relatives, the great apes (chimpanzees, bonobos, gorillas, and orangutans) [10-17] (Figure 1). Nevertheless, technological limitations restricted the types of lipids that could be quantified in these early studies. More comprehensive measurements are important for testing hypotheses that changes in lipid metabolism influenced the evolution of numerous traits in the human lineage, including those relevant to cognition and cardiovascular health [18-27].

**Figure 2:**
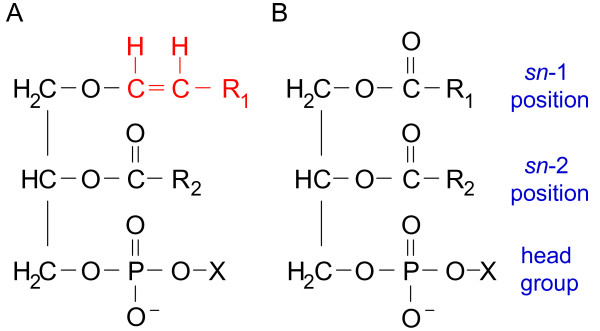
**Basic plasmalogen structure**. (A) Plasmalogens are glycerophospholipids characterized by the presence of a vinyl-ether linkage at the *sn*-1 position and an ester-linkage at the *sn*-2 position. R1 and R2 represent straight-chain carbon groups. At the *sn*-1 position, the chemical moiety highlighted in red is an alkenyl group, which we use to measure plasmalogen abundance and molecular composition. These alkenyl groups are most commonly derived from C16:0, C18:0, or C18:1 fatty alcohols. The *sn*-2 position of plasmalogens is occupied typically by polyunsaturated fatty acids. X represents the head group, typically ethanolamine or choline for plasmalogens. In contrast, (B) diacylglycerophospholipids have ester-linkages at their *sn*-1 and *sn*-2 positions. As above, R1 and R2 represent straight-chain carbon groups and X represents the head group. Adapted from reference [31].

**Figure 3:**
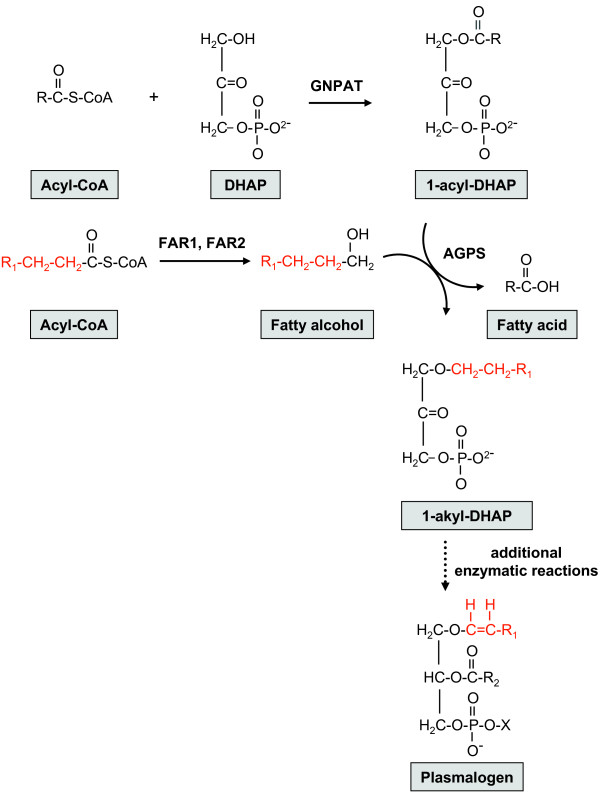
**Initial steps of plasmalogen biosynthesis**. Plasmalogen biosynthesis requires enzymes located in peroxisomes and the endoplasmic reticulum. The initial step, catalyzed by the peroxisomal enzyme dihydroxyacetonephosphate acyltransferase (GNPAT), is the acylation of dihydroxyacetone phosphate (DHAP) at the *sn-1 *position to form 1-acyl-DHAP [34]. Fatty acyl-CoA reductase (FAR1 and/or FAR2), located on the cytoplasmic aspect of the peroxisomal membrane, catalyzes the NADPH-dependent conversion of a fatty acyl-CoA to the corresponding fatty alcohol [33]. The fatty alcohol enters the peroxisome and displaces the *sn-1 *fatty acid in a reaction catalyzed by alkyl-dihydroxyacetone phosphate synthase (AGPS), resulting in formation of 1-akyl-DHAP, which has an ether linkage [35]. Subsequent enzymatic steps occur outside the peroxisome, including the reduction of alkyl-dihydroxyacetone phosphate to alkyl-glycerophosphate, addition of an acyl group in the *sn-2 *position, desaturation of the alkyl group to an alkenyl group, and addition of choline or ethanolamine head groups [31]. PEX7 is required for the import of AGPS into peroxisomes and *PEX7 *mutations, which are the primary cause of RCDP, lead to impaired plasmalogen biosynthesis.

Plasmalogens are ether-phospholipids present in mammalian plasma and intracellular membranes [28-31]. They comprise about 20% of the phospholipid mass in humans and chemically differ from more abundant glycerophospholipids as well as other ether phospholipids by the presence of a vinyl ether bond at the *sn-1 *position [30-35]. Plasmalogens can differ based on the chemical group at the *sn*-1 position (primarily derived from C16:0, C18:0, and C18:1 fatty alcohols) and the *sn*-2 position (commonly arachidonic acid or docosahexaenoic acid) as well as their head group [36-39] (Figure 2). The majority of plasmalogens in mammalian tissues bear ethanolamine (1-O-alk-1'-enyl-2-acyl-*sn*-glycerophosphoethanolamine, plasmenylethanolamine) or choline-linked head groups (1-O-alk-1'-enyl-2-acyl-*sn*-glycerophosphocholine, plasmenylcholine) [30]. Plasmalogens are especially enriched in nervous and cardiac tissues as well as the spleen and cells of the immune system [30]. Genetic deficiency or cellular mislocalization of one of the two peroxisomal enzymes that initiate plasmalogen biosynthesis, GNPAT and AGPS, results in the severe disorder rhizomelic chondrodysplasia punctata (RCDP) [40-42] (Figure 3). The clinical phenotypes of human RCDP patients [40-47] and genetically engineered mouse models [32] indicate that plasmalogens are necessary for normal neurological, skeletal, visual, respiratory, and reproductive functions. Decreased brain tissue plasmalogen levels also have been associated with Alzheimer Disease [48-55], X-linked adrenoleukodystrophy [56,57], and Down syndrome [58].

Here, we address the possibility that plasmalogens influence species-specific traits among humans and great apes. We compared red blood cell (RBC) plasmalogen levels and cellular rates of plasmalogen biosynthesis in cohorts of humans and great apes. Human vegan RBC data were used to assess the effects of meat and dairy consumption, which are relevant to comparisons with the mostly plant-eating great apes [24]. Overall, we observed that human RBC plasmalogen profiles differed from those of the great apes and provide indirect evidence that this extends to other tissues, which could affect functions relevant to the evolution of these species.

## Materials and methods

### Cohort for RBC lipid profiling

Blood samples from adult humans with Western diets were collected from healthy individuals attending an international conference. Blood samples from adult humans on vegan diets for over one year were collected in conjunction with a blood donor center. Appropriate Institutional Review Board (IRB) approval from the University of Southern California and Johns Hopkins Medicine was obtained for all human subjects research. Chimpanzee blood samples were collected at the Alamogordo Primate Facility. All chimpanzees took part in daily enrichment activities to maintain psychological well-being. The chimpanzees were maintained in accordance with the Guide for the Care and Use of Animals (U.S. Dept. of Health and Human Services, Public Health Service, Bethesda, MD., 1996). The APF and its program were fully accredited by the Association for Assessment and Accreditation of Laboratory Animal Care, International (AAALAC). Other great ape bloods were collected at the Zoological Society of San Diego (ZSSD). The gender and ages of all blood donors are provided in Additional File 1. Great ape diets contain fresh fruits, vegetables, and nutritional biscuits.

### RBC lipid profiling

Whole blood samples were collected from fasting subjects and stored in EDTA blood collection tubes. RBCs were collected by centrifugation, washed twice with physiological saline, transferred to freezer vials, flushed with nitrogen, and stored at -80°C until analysis. RBCs were thawed briefly before 100 μl aliquots were taken for analysis of the total lipid fatty acid content as their DMAs by capillary GC with flame ionization detection [59]. Processed data are provided in Additional File 2.

### Primary fibroblast cultures

Great ape dermal fibroblasts were obtained from the ZSSD while human dermal fibroblasts were obtained from the Coriell Institute for Medical Research or the Kennedy Krieger Institute. All individuals are thought to be unrelated. The gender, age, and biopsy site of all fibroblast donors and corresponding biochemical analyses are provided in Additional File 3. Fibroblasts were cultured as previously described [60].

### Plasmalogen biosynthesis in cultured fibroblasts

Assays were performed as previously described [61]. They are based on the incorporation of ^14^C-hexadecanol and ^3^H-hexadecyl-glycerol into plasmalogens. All processed data are provided in Additional File 4.

### Statistical considerations

We analyzed all data on the log2 scale. We used analysis of variance (ANOVA) to compare average blood lipid data across humans and great apes. Heterogeneity *P*-values are reported for the test that the mean level is different in at least one of great apes groups, and Wald *P*-values for tests comparing the average level in individual non-human primate groups to humans. Under ANOVA, statistical tests use an estimate of within-group variation from all samples. Due to the unbalanced group sizes, this estimate is driven by the variation in humans and in chimpanzees. Box plots showing the distribution of the data suggest that the equal-variance assumption is appropriate. For gene expression studies, we only considered data from oligonucleotide probes predicted to be perfectly matched to both genomes and only assigned gene expression scores to probe sets containing at least four such probes [62]. We tested for differential expression using moderated t-tests and F-tests, as described [63]. All analyses were done using the R programming language. All processed data are provided in Additional File 5.

## Results

RBC plasmalogen levels are used in diagnostic tests for human disorders involving impaired plasmalogen biosynthesis [64,65]. To conduct a cross-species comparison of cellular plasmalogen profiles, we measured RBC plasmalogen levels in a cohort of humans with Western diets (N = 120), humans with vegan diets for over one year (N = 16), chimpanzees (N = 46), bonobos (N = 4), lowland gorillas (N = 7), and Sumatran orangutans (N = 3) (Additional File 1). We measured the levels of the C16:0, C18:0, and C18:1 chemical moieties most commonly present in the *sn*-1 position of plasmalogens based on their dimethyl acetal (DMA) derivatives produced during sample preparation (Methods) (Additional File 2). Total plasmalogen levels were estimated based on the sum of C16:0 DMA, C18:0 DMA, and C18:1 DMA levels. Plasmalogen composition was analyzed based on the levels of specific DMA derivatives.

### Human and great ape RBC plasmalogen levels

**Figure 4:**
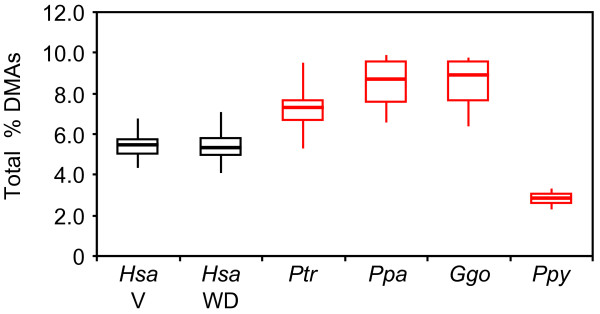
**Total plasmalogen levels in human and great ape RBCs**. Modified box plots representing the percentage of total plasmalogens relative to total fatty acids (Y-axis) from RBCs obtained from humans and great apes are shown. Median, quartile 1, quartile 3, minimum, and maximum values are provided. Total plasmalogens are represented as the sum of the percentage of the C16:0 DMA, C18:0 DMA, and C18:1 DMA derivatives of the chemical moieties present in the *sn*-1 position of plasmalogens relative to total fatty acids. The species (*Hsa*: human; *Ptr*: chimpanzee (N = 46); *Ppa*: bonobo (N = 4); *Ggo*: gorilla (N = 7); *Ppy*: orangutan (N = 3), human diet (V: vegan diet (N = 16), WD: western diet (N = 120) is provided on the X-axis. Animal cohort data showing a significant difference (*P *< 0.05) relative to both human diet groups (red), vegans alone (green), or Western diet alone (blue) are color-coded as indicated.

Total RBC plasmalogen levels differed in the human Western diet (WD) and vegan groups relative to the great apes (ANOVA *P *< 1 × 10^-4 ^for both comparisons) (Figure 4). Both human diet groups had lower total plasmalogen levels relative to chimpanzees, bonobos, and gorillas (≥1.3-fold, *P *< 1 × 10^-6 ^for all six comparisons). In contrast, total RBC plasmalogen levels in both human diet groups were elevated relative to orangutans (1.9-fold, *P *< 1 × 10^-10 ^for both comparisons).

### Human and great ape RBC plasmalogen composition

**Figure 5:**
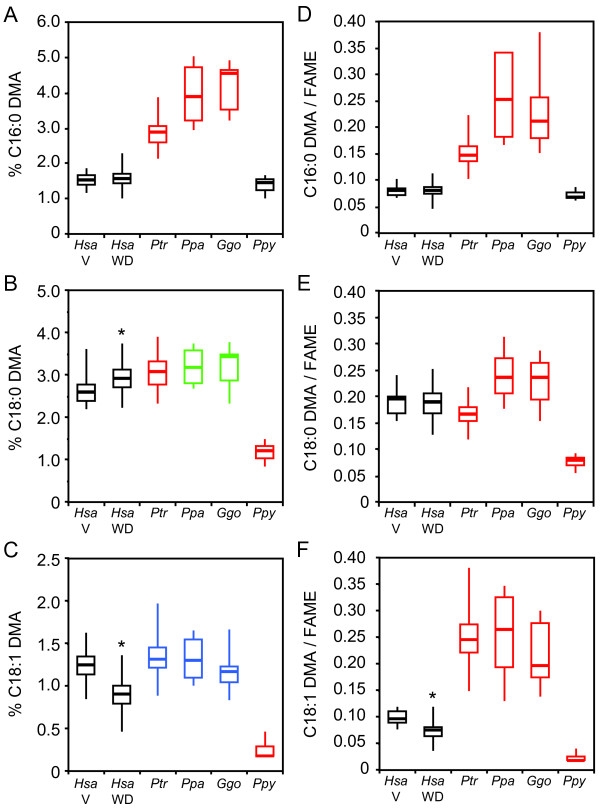
**Human and great ape RBC plasmalogen composition**. On the left most panels, we provide modified box plots representing the percentage of the DMA derivatives of (A) C16:0, (B) C18:0, and (C) C18:1 chemical moieties present in the *sn*-1 position of plasmalogens relative to total fatty acids (Y-axis) in RBCs. On the rightmost panels, we provide modified box plots representing the ratio of the abundance of (D) C16:0, (E) C18:0, and (F) C18:1 chemical moieties present in the *sn*-1 position of plasmalogens with respect to their cognate fatty acids (Y-axis) in RBCs. These are reported as DMA/FAME ratios since the vinyl either-linked groups and cognate fatty acids are converted to dimethyl acetyl (DMA) and fatty acid methylester (FAME) derivatives, respectively, after RBC sample processing. In Panel F, C18:1 FAME levels represent the sum of C18:1 (n-5), C18:1 (n-7), and C18:1 (n-9) FAMEs. C18:1 (n-5) FAME levels could not be measured in one orangutan, consistent with its trace abundance in the other two orangutans. The numbers and identities of RBC donors (X-axis) is the same as in Figure 2. Animal cohort data are color-coded as indicated in Figure 4. The star in Panels B, C, and F indicate that the DMA level or DMA/FAME ratio in the human vegan and Western diet cohort differ (*P *< 0.05).

The C16:0 DMA, C18:0 DMA, and C18:1 DMA levels from both human diet groups differed from those of the great apes (ANOVA *P *< 1 × 10^-15 ^for all six comparisons) (Figure 5A-C). Both human diet groups had substantially lower C16:0 DMA levels relative to chimpanzees, bonobos, and gorillas (≥1.9-fold, *P *< 1 × 10^-15 ^for all six comparisons) (Figure 5A), but only mildly lower C18:0 DMA levels relative to chimpanzees (≤1.2-fold, *P *< 0.05) (Figure 5B). Vegans also had lower C18:0 DMA levels relative to bonobos and gorillas (1.2-fold, *P *< 0.05 for both comparisons). Both human diet groups had markedly higher C18:0 DMA and C18:1 DMA levels relative to orangutans (≥2.3-fold, *P *< 1 × 10^-12 ^for both comparisons). The human WD group also had lower C18:1 DMA levels relative to chimpanzees, bonobos, and gorillas (≥1.3-fold, *P *< 1 × 10^-3 ^for all three comparisons) (Figure 5C).

To address the fact that the identity of the moiety at the *sn*-1 position of plasmalogens is influenced by the cellular levels of related fatty acid levels, we normalized C16:0, C18:0, and C18:1 DMA levels with respect to those of their cognate fatty acids. Since fatty acids are converted to methyl ester (FAME) derivatives after sample processing, we report these as DMA/FAME ratios (Figure 5D-F). C16:0, C18:0, and C18:1 DMA/FAME ratios in both human diet groups differed from those of the great apes (ANOVA *P *< 1 × 10^-6 ^for all six comparisons). Relative to both human diet groups, the chimpanzees, bonobos, and gorillas had higher C16:0 and C18:1 DMA/FAME ratios (≥1.9-fold, *P *< 1 × 10^-9 ^for all six comparisons), but the orangutans had lower C18:0 and C18:1 DMA/FAME ratios (≥2.5-fold, *P *< 1 × 10^-14 ^for all four comparisons) (Figure 5E,F). Also relative to both human diet groups, the C18:0 DMA/FAME ratio was lower in chimpanzees (≤1.1-fold, *P *< 0.05 for both comparisons), but higher in bonobos and gorillas (1.2-1.3-fold, *P *< 0.05 for all four comparisons).

### RBC plasmalogen levels and composition relative to human and chimpanzee gender

Our cohort provided adequate statistical power to screen for possible sexual dimorphism in human (WD group) and chimpanzee plasmalogen levels and composition. No significant (>1.1-fold, *P *< 0.05) sex-specific differences in total DMA levels, specific DMA levels, or DMA/FAME ratios were found within this cohort of chimpanzees or humans. Nevertheless, human males had a slightly higher (1.1-fold, *P *< 0.05) C16:0 DMA/FAME ratio relative to human females.

### Human RBC plasmalogen levels in the vegan and Western diet (WD) groups

The human vegan and WD groups showed no significant differences in total plasmalogen or C16:0 DMA levels or their C16:0 and C18:0 DMA/FAME ratios (*P *> 0.05) (Figures 4 and 5). Nevertheless, C18:0 DMA levels were slightly higher (1.1-fold, *P *< 0.01) for the WD group. Vegans had higher C18:1 DMA level and C18:1 DMA/FAME ratios relative to the WD group (1.3-1.4-fold elevated, *P *< 1 × 10^-2 ^for both comparisons).

### Cellular rates of peroxisomal plasmalogen biosynthesis

**Figure 6:**
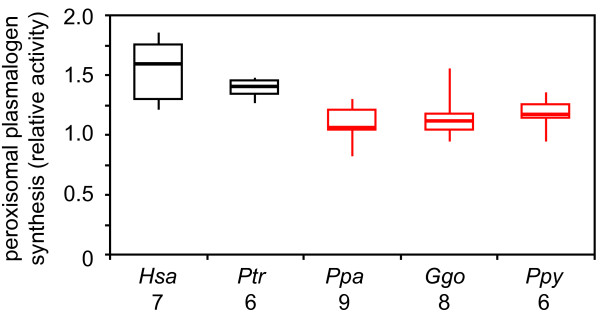
**Rates of peroxisomal plasmalogen biosynthesis in human and great ape cultured fibroblasts**. The relative rates of the peroxisomal relative to endoplasmic reticulum (ER) mediated steps of plasmalogen biosynthesis in cultured skin fibroblasts are provided in modified box plots. Larger ratios indicate increased activity of the peroxisomal relative to ER components of plasmalogen biosynthesis. The number of cultures analyzed is provided on the X-axes. Animal cohort data are color-coded as indicated in Figure 4.

Cultured skin fibroblasts are used in clinical settings to measure cellular plasmalogen levels, rates of plasmalogen synthesis, and other peroxisomal functions [61,66-68] (Additional File 3). We used this system to show that the rates of the peroxisomal component of plasmalogen biosynthesis, recently suggested to regulate the entire biosynthetic pathway [69], differed in human relative to great ape cultured skin fibroblasts (ANOVA *P *= 2.2 × 10^-4^) (Figure 6) (Additional File 4). These rates were greater in human relative to bonobo (1.4-fold, *P *= 3.6 × 10^-6^), gorilla (1.3-fold, *P *= 6.7 × 10^-4^), and orangutan (1.3-fold, *P *= 2.8 × 10^-3^) skin fibroblasts. No differences in the rates of peroxisomal plasmalogen biosynthesis were found between human and chimpanzee skin fibroblasts.

### Comparative transcriptomics of peroxisomal plasmalogen biosynthetic pathways

**Figure 7:**
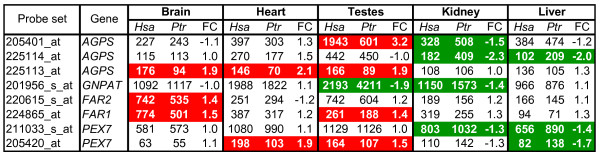
**Differential expression of genes related to ether phospholipid metabolism**. We provide data from the reanalysis of previously published Affymetrix GeneChip U133v2.0 expression profiles of human and chimpanzee tissues [70]. We used moderated F-tests (false discovery rate (FDR)) adjusted using the Benjamini and Hochberg approach) to test for differences in the distributions by species for the five tissues. Only probe sets with a ≤5% FDR are shown. Geometric mean expression scores are provided for human (*Hsa*) and chimpanzee (*Ptr*) tissues. FC: fold change of *Hsa *relative to *Ptr *expression. Transcript data are highlighted in red (up in human) or green (up in chimpanzee) if they show ≥1.2 FC (absolute value) with a moderated t-test *P*-value ≤0.05 after Bonferroni correction. Affymetrix probe sets IDs are provided.

We re-analyzed existing gene expression data [70] from human and chimpanzee livers, brains, kidneys, heart, and testes to begin to explore the possibility that cross-species differences in plasmalogen composition occur in these tissues (Methods). We focused on genes described in Figure 3 that are specifically involved in the initial steps of plasmalogen biosynthesis [30,69] and identified differentially expressed genes (DEGs), as stated in the Figure 7 legend. Cross-species DEGs varied according to the tissue examined (Figure 7 and Additional File 5). For example, all the DEGs in brain (*AGPS*, *FAR1*, and *FAR2 *transcripts) and heart (*AGPS *and *PEX7 *transcripts) were more abundant in humans relative to chimpanzees. In contrast, all the DEGs in kidney (*AGPS*, *GNPAT*, and *PEX7 *transcripts) and liver (*AGPS *and *PEX7 *transcripts) were more abundant in chimpanzees relative to humans. In testes, humans showed 3.2-fold increases in *AGPS*, but 1.9-fold reductions in *GNPAT *transcript levels, relative to chimpanzees. *FAR1 *and *PEX7 *transcripts were also more abundant in human relative to chimpanzee testes.

## Discussion

Plasmalogens have had a complex evolutionary history with differing biosynthetic pathways in aerobic and anaerobic organisms [71]. Their levels are also known to vary among mammals. For example, human, rat, and guinea pig plasmalogen levels differ in multiple tissues [72] Furthermore, the higher tissue plasmalogen levels in long relative to short-lived rodents has been suggested confer a lower susceptibility to oxidative membrane damage and contribute to an extended lifespan [29]. Considering how plasmalogen deficits impact human and rodent health, they represent intriguing candidate molecules that could contribute to developmental, physiological, and cognitive differences among humans and great apes.

We observed striking differences in human and great ape RBC plasmalogens. For example, both human diets groups had lower RBC total plasmalogen levels relative to the African great apes (chimpanzees, bonobos, and gorillas), but higher RBC total plasmalogen levels relative to orangutans (Figure 4). Most notably, RBC plasmalogens with a C16:0 moiety at their *sn*-1 position were considerably more abundant in African great apes relative to both human diet groups and orangutans, which had similar levels (Figure 5 A, D). The diverse plasmalogen profiles in our cohort highlight the importance of including all four great ape species in comparative studies with humans in order to avoid spurious inferences. Adaptations that occurred in each lineage, especially those related to their historic diets, could make it challenging to infer ancestral states.

We favor the hypothesis that cross-species differences in plasmalogen metabolism are responsible for the distinctions between human and great ape RBC plasmalogen profiles. Although open to discussion [30], recent evidence suggests that plasmalogen biosynthesis in regulated by the peroxisomal component of this pathway [69]. In this regard, we observed small differences in the rates of peroxisomal plasmalogen biosynthesis in human relative to other great ape skin fibroblasts (Figure 6). These studies should be replicated and expanded in order to examine rigorously intraspecies variation, plasmalogen composition, and the ER component of plasmalogen biosynthesis. Likewise, cross-species differences in plasmalogen turnover rates could have influenced our results. In this regard, plasmalogen-selective phospholipase A2 (PLA_2_) can release the *sn*-2 fatty acid to yield lysoplasmalogens [30,31,73], which can be degraded by lysoplasmalogenases or acylated to produce intact plasmalogens [73-78]. Although lysoplasmalogen levels can increase after stress [79,80], they are generally thought to be of minor abundance relative to intact plasmalogens. To the best of our knowledge, RBC plasmalogen half-lives have not been reported, but the turnover rates of RBC membrane phospholipids is relatively slow compared to other organs, such as liver [64]. Nevertheless, rat brain ethanolamine and choline plasmalogens have short, but different, half-lives [31,49,81].

Another candidate explanation for our observations is that humans and great apes differ in their ability to derive plasmalogens from dietary sources. Plasmalogens are present in substantial amounts in meat and fish products commonly found in Western diets [82]; however, they are rarely found in plants [83,84]. Wild great apes are mainly plant-eating and our captive population is not exposed to significant amounts of meat products based on their diets, which we previously showed have very low levels of phytanic acid, a sensitive biomarker of ruminant fats, dairy, and certain fish products [24]. We found that the human WD and vegan groups had quite similar RBC plasmalogen levels and composition (Figures 4 and 5). Even the moderately higher C18:1 DMA levels in the vegan group did not alter the conclusions from cross-species comparisons involving C18:1 DMA/FAME ratios. This is in broad agreement with other studies wherein RBC plasmalogen levels did not change in a limited number of humans on corn oil, triolein, or butter fat-enriched diets [85,86]. Nevertheless, modest increases in C18:1 ethanolamine plasmalogen RBC levels occurred in subjects on a triolein-enriched diet [85].

Animal models have produced a complex picture of how diet influences tissue plasmalogens [87-105]. Rats consuming unnaturally high levels of plasmalogens (>10% by weight) showed elevated blood plasma and liver plasmalogen levels; however, their RBC, skeletal muscle, brain, kidney, lung, or adipose tissue plasmalogen levels were not significantly altered [89]. Alkylglycerols (AGs) are natural products present in human and other mammalian diets that can be incorporated into plasmalogens and influence the identity of the chemical group at the *sn*-1 position [88,106]. Nevertheless, AG consumption only has a minor influence on total plasmalogen levels in tissues from whole animals [107,108]. While the AG dietary levels are not well-defined, one report [88] suggests that adults can daily consume 10-100 mg of batyl alcohol, a type of AG especially abundant in shark liver oil [109]. Dietary fatty alcohols obtained from certain vegetables and fish could also affect the identity of the chemical group at the *sn*-1 position [30]. Nevertheless, the strong similarities between the vegan and WD group plasmalogen profiles suggest that differences in AG and/or fatty alcohol consumption did not significantly influence our results.

Age is another factor reported to affect RBC plasmalogens. In a cohort of younger (25-39 years old) and older (average 79 years old) humans, RBC 16:0 DMA levels did not correlate with age, but small age-related decreases in RBC C18:0 DMA levels were observed (0.04% per decade) [110]. Nevertheless, although our human WD group is older on average than our vegan group (51 vs 35 years), the former has slightly elevated C18:0 DMA levels relative to the latter. Considering cross-species differences in life expectancies [111-115], the age composition of our human and great ape cohorts is relatively uniform (Additional File 1).We also note that the C16:0 and C18:0 DMA/FAME ratios in both our human diet groups are in good agreement with those reported for neonates and children [116]. While we cannot preclude that donor age influenced our results, we suggest that these effects are minor compared to those related to donor species.

In efforts to explore the possibility that differences in plasmalogen levels and composition extend to other tissues, we found that key genes specifically involved in their biosynthesis were differentially expressed in human and chimpanzee organs (Figure 7). The higher abundance of *FAR1*, *FAR2*, and *AGPS *transcripts in human relative to chimpanzee brain is especially interesting since plasmalogens comprise almost 30% of the glycerophospholipids in the adult human brain and up to 70% of human myelin sheath ethanolamine glycerophospholipids [49]. Our gene expression analyses suggest the possibility of cross-species differences in brain plasmalogen levels and/or composition that could have a special impact on the brain's white matter, which is critical for cognitive processes [117]. The potential significance of white matter volumes in human and non-human primate brain evolution has been discussed [118-122]. The proposed antioxidant properties of plasmalogens [30,31] could be especially important in human brains, which have higher metabolic demands than those of chimpanzees and other non-human primates (NHPs) [123]. The DEGs in testes are intriguing given male infertility in mice with plasmalogen defects [124], variation in mammalian spermatozoa phospholipid composition [125], and differences in human and great ape male reproductive systems [126]. Since comparatively less is known about the role of plasmalogens in kidney and liver function, we simply note multiple instances of higher plasmalogen biosynthesis-related transcript levels in chimpanzees relative to humans.

Although we cannot make definitive conclusions about the physiological ramifications of the cross-species differences observed in our studies, specific clinical phenotypes have been associated human RBC plasmalogen levels and composition [127-133]. For example, it was reported that hyperlipidemic individuals had 20% reduced RBC ethanolamine plasmalogen levels relative to healthy controls [127]. Furthermore, significant inverse correlations have been found between human RBC C16:0 DMA/FAME ratios and total cholesterol, triglycerides, body fat mass, and glycosylated hemoglobin [128]. Reduced RBC C16:0 and C18:0 DMA/FAME ratios were also associated with human malnutrition [128]. It is formally possible that the observed differences in plasmalogen levels and composition relate to differences in cholesterol regulation in humans, captive chimpanzees, and possibly other captive NHPs [21]. Furthermore, it is tempting to speculate that low RBC total plasmalogen levels in orangutans relate to the basal metabolic rate of this species, which is lower than those of humans and chimpanzees [134,135]. Given their antioxidant properties, an increased plasmalogen levels could be beneficial for species with higher metabolic rates.

Although caution must be taken when using human medical data to interpret genetic and biochemical differences among human and NHPs, this approach is useful for generating hypotheses, which can be tested in appropriate cell culture and/or laboratory animal models [136-140]. It also will be necessary to measure the levels of distinct plasmalogens in multiple human and NHP cell types and tissues in order to refine and test these hypotheses. Comparative analyses of the human and NHP nervous, cardiovascular, and reproductive systems are especially relevant given the phenotypes of humans and mice with severely impaired plasmalogen biosynthesis. The application and further development of lipidomic tools and technologies will play a vital role in this process [141].

## Conclusions

We observed robust differences in RBC total plasmalogen levels and composition among humans and great apes. Our favored hypothesis is that cross-species differences in plasmalogen metabolism are responsible for the distinctions between human and great ape RBC plasmalogen profiles. In contrast to these species-related differences, the human diets studied had lesser impacts on RBC plasmalogen composition and none on total plasmalogen levels. Likewise, we did not observe robust sex-specific differences in human or chimpanzee RBC plasmalogen levels or composition. Gene expression profiles raise the possibility that other human and great ape cells and tissues differ in plasmalogen levels, which could influence developmental, physiological, and cognitive functions relevant to the evolution of these species.

## Competing interests

The authors declare that they have no competing interests.

## Authors' contributions

ABM, and SJS carried out the biochemical analyses in this project. ABM, PAW, and HWM were involved in the design and conception of the peroxisome components of this project. KR maintained and conducted genetic and biochemical analysis on human and great ape cells. KDS conducted statistical analyses of all biochemical and gene expression data. RL and JJE provided characterized chimpanzee blood samples and diet information. OAR provided characterized great ape cells, blood samples, and diet information. OAR, JJE and RL were involved in the design and conception of the great ape components of the project. JGH was involved in the overall design and conception of the project, statistical analysis of all data sets, and wrote the manuscript with the help of all authors.

All authors have read and approved the final manuscript.

## Supplementary Material

Additional file 1 **Composition of blood donor cohort**. A summary of the numbers, ages, and sex of blood donors is provided. Click here for file

Additional file 2 **Red blood cell plasmalogen composition from all donors**. Relative levels of plasmalogens for all RBC donors are provided. Click here for file

Additional file 3 **Skin fibroblast cultures used for plasmalogen analysis**. A summary of donor sex and age and anatomical source of the skin fibroblasts is provided. Click here for file

Additional file 4 **Rates of peroxisomal plasmalogen biosynthesis in cultured dermal fibroblasts**. The rates of peroxisomal plasmalogen biosynthesis from all individual fibroblast cultures are provided. Click here for file

Additional file 5 **Detailed summary of gene expression data for cross-species comparisons**. More complete gene expression data summary statistics relevant to Figure 7 are provided. Click here for file
